# Pectin and Its Beneficial Effect on Health: New Contributions in Research and the Need to Increase Fruits and Vegetables Consumption—A Review

**DOI:** 10.3390/ijms26146852

**Published:** 2025-07-17

**Authors:** Luis Valladares, Fernando Vio

**Affiliations:** 1 Human Nutrition Unit, Institute of Nutrition and Food Technology (INTA), University of Chile, Santiago 7830490, Chile; lvallada@inta.uchile.cl; 2 Public Nutrition Unit, Institute of Nutrition and Food Technology, University of Chile, Santiago 7830490, Chile

**Keywords:** pectin, starch, glycemic response, digestion, diabetes, obesity, cardiovascular diseases, dietary fiber, fruits and vegetables

## Abstract

The beneficial effect of consuming fruits and vegetables in the prevention of chronic non-communicable diseases and healthy aging is well known. This is attributed to food and vegetable antioxidant and fiber content. The aim of this publication is to communicate the results of recent research on pectin in humans, to propose an increased consumption of fruits and vegetables, or their possible use as a food supplement. A comprehensive narrative review was conducted considering recent publications on pectin. The description of starch, pectin, the physicochemical changes caused by pectin, and the effect of pectin on the activity of amylase are reported. Dietary fiber and gut microbiota in human health are also described, with the production of saturated fatty acids with fewer than six carbon atoms. Finally, health effects such as anti-hyperglycemic and anti-hyperlipidemic activities, preventing and controlling obesity and heart disease, are analyzed, as well as other health effects in tumors, the gastrointestinal tract, and immunity. Considering the beneficial effects of pectin in health and the low consumption throughout the world, it is recommended to promote the consumption of fruits and vegetables to increase pectin intake in the human diet.

## 1. Introduction

The beneficial effect of consuming fruits and vegetables in the prevention of chronic non-communicable diseases and healthy aging is well known. The scientific basis for the concept of encouraging fruit and vegetable (F&V) consumption stems from observational studies that intensified from the 1980s onwards, with publications on its effect on cancer, such as those by Willett, 1990 [1], Negri et al., 1991 [2], Steinmetz and Potter, 1991 [3] and Ziegler, 1989 [4], 1991 [5].

In the case of cardiovascular diseases (CVD), the studies were later, with evidence from the Stephen study in 1993 [6], which found that flavonoids in tea, onions, and apples decreased the risk of CVD mortality. The Framingham study (1995) [7] in 832 subjects with 20 years of follow-up showed that the consumption of F&V decreased the risk of stroke. Another study, the NHANES Health and Nutrition Study (USA, 2002) [8] in 9608 subjects and 19 years of follow-up showed that F&V decreases the risk of all-cause mortality from CVD.

This has been recognized by the World Health Organization (WHO) since 2002 [9] and then confirmed by multiple studies. In addition, it has been demonstrated that those who eat more than 400 g of fruits and vegetables per day have less mortality [10]. In general, this is attributed to food and vegetable antioxidant content as well as fiber content.

The aim of this publication is to communicate the results of recent research on pectin, a significant compound of fruit and vegetables, and its effects on health, with the aim of proposing a greater consumption of F&V, or its possible use as a food supplement or supplement once isolated from its vegetable support.

## 2. Effect of Pectin on Starch Digestion

### 2.1. Starch

Starch is the main carbohydrate of the human diet and therefore contributes most of the energy requirement for the world population [11]. The starch molecules it is made up of amylose and amylopectin, with typical levels of 20–30% and 70–80%, respectively. Amylose possesses a linear structure of α-D-glucose units joined by the α-1-4-glycosidic bond, while amylopectin possesses a branched structure with α-1-4 as well as α-1-6 glycosidic linkages. Starch digestibility is one of the relevant criteria for starch quality, and depending on its digestibility, ingested starch can exert different effects on health [12]. In humans, the degradation of starch begins with salivary and pancreatic α-amylase in the small intestine and is completed by the action of the brush-border enzymes maltase–glucoamylase, and sucrose–isomaltase [13]. Per nutritional benefits and according to the rate of digestion, starch is classified into three different fractions: rapidly digestible starch (RDS), slowly digestible starch (SDS), and resistant starch (RS) [14]. RDS is the starch fraction that causes a rapid increase in blood glucose level after ingestion. SDS is the starch fraction that is digested slowly and delivers a slow and sustained release of blood glucose. RS is the starch portion that resists digestion in the small intestine and ferments in the large intestine [15]. Consistent evidence shows a positive relationship between postprandial hyperglycaemia in obesity [16], type 2 diabetes mellitus (T2DM) [17,18], and CVD [19]. This indicates that regulating the digestion of starchy foods and the absorption of glucose is essential for maintaining blood glucose homeostasis, and it is an interesting option for the prevention and treatment of metabolic disorders. The most relevant factors in starch digestibility in foods are related to the physicochemical characteristics of starch, including the amylose–amylopectin (AP) ratio, amylase content, AP chain length distribution, granule size, and crystallinity characteristics. Higher amylose content reduces starch degradation, as the tightly packed amylose structure, stabilized by hydrogen bonds, is highly resistant to enzyme hydrolysis [20,21]. Many studies have reported that starch becomes more digestible as granule size decreases because smaller particles provide a larger surface area, allowing digestive enzymes to interact more effectively with the starch [22]. Starch rich in short-chain amylose exhibits poor digestibility because short amylose chains impact the starch gel characteristic that reduces enzyme susceptibility [23]. The crystallinity characteristics of starch include A-type (cereals), B-type (tubers), and C-type (legumes). In general, type B/C starches have a much slower digestion rate than type A starches, since type A starches have surface pores that allow faster diffusion of digestive enzymes into the granules [21,24].

### 2.2. Pectin

Pectin is a soluble dietary fiber found in the primary cell wall and intracellular layer of plant cells, mainly in fruits and vegetables, such as apples, bananas, oranges, lemons, blueberries, grapefruit, carrots, fava beans, and tomatoes [25,26]. Pectins are complex heteropolysaccharides that include at least 17 kinds of monosaccharides. Relevant to understanding the biological activity of pectins is that pectin does not have a precise structure, and its molecular arrangement depends on the source, food matrix, fruit ripening, and other environmental factors [25,26].

**Figure 1:**
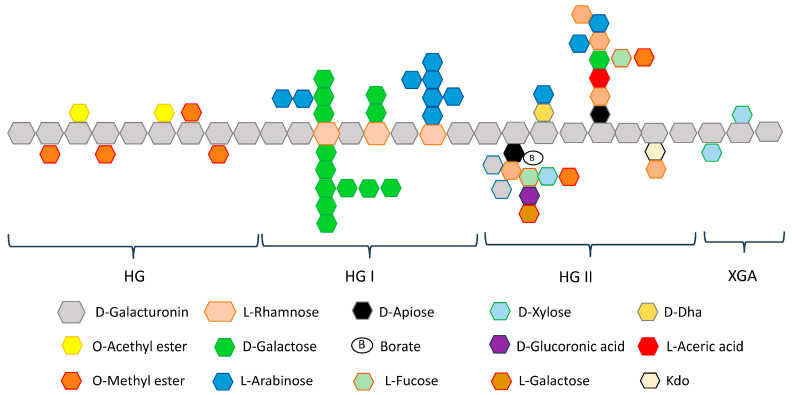
Schematic pectin structure. Adapted from [28].

Pectin is generally divided into four distinct structural categories (Figure 1), including homogalacturonan (HG), xylogalacturonan (XGA), rhamnogalacturonan I (RG-I), and rhamnogalacturonan II (RGII) [27]. The main backbone residue of pectin is α-1-4-D-galacturonic acid (GalA). HG consists exclusively of GalA units in which some of the carboxyl groups are esterified with methanol and/or acetyl groups. Based on the degree of esterification (DE), pectins can be divided into low methoxyl pectins (<50%) and high methoxyl pectins (>50%). The degree of methylation, expressed as the ratio of methyl-esterified carboxyl groups to the total amount of galacturonic acid units, is one of the key parameters related to gelling capability [28].

Dietary fiber is another important component of a healthy diet. However, as recently indicated by Bai and Gilbert [29], pectin compared to other dietary fibers, has some advantages in regulating starch digestion, including (1) high frequency in fruit and vegetables, (2) reliable safety, and abundant health benefits, (3) the complex molecular structure of pectin’s is versatile, with the potential to create tailor-made properties. This allows us, in part, to understand the results of the numerous studies of the last decades, where a diet with an inadequate fiber intake is negative for human health [30].

Although the mode of action of dietary fiber in the consumer’s body is not fully understood, much knowledge has been gained in the last lustrum, especially about the molecular mechanism of pectin [29,31].

### 2.3. Physicochemical Changes Caused by Pectin

The capacity of pectin to regulate both starch digestion and postprandial hyperglycaemia may be achieved by different mechanisms: inhibiting the activity of some enzymes, modifying the viscosity of the digest, and regulating metabolic pathways.

In solution, the hydrophilic chains of pectin molecules become hydrated and will entangle with each other to form complex networks [32] that macroscopically increase the viscosity of the solution. The physicochemical environment of the digest in vivo is complex due to (1) the diversity of compounds in the gastrointestinal tract; (2) ion composition and variation in pH values at different phases of the GI tract; (3) physiological responses stimulated by nutrient ingestion.

Several in vivo studies [33,34,35,36] have shown that the addition of pectin can increase the viscosity of the digest. As a result, during passage through the gastrointestinal tract the blending of digest, interaction between enzymes and substrates, diffusion of substrates and digestive enzymes [34,37,38] and the movement of chime [12,39,40] are slowed down, resulting in less gelatinization of starch, less digestion of starch and less nutrient absorption are all slowed down, resulting in less digestion of starch and less nutrient absorption. It has been suggested that digest mixtures could form gels because of pectin, which also leads to slowed movement in the gastrointestinal tract.

### 2.4. Impact of Pectin on the Activity of Enzymes Amylases

After ingestion, starch is digested to glucose sequentially by salivary α-amylase, pancreatic α-amylase, and intestinal brush border enzyme, and the glucose is then absorbed by enterocytes and released into the bloodstream [41]. As mentioned above, postprandial glucose has been implicated in the development of obesity [16], T2DM [17,18], and CVD [19,42]. Therefore, the inhibition of the activity of α-amylases has long been used as an effective approach to control postprandial blood glucose levels and in vivo starch digestion [33,43]. Consequently, finding new amylase inhibitors, especially those found in ordinary food, is of ongoing interest [29,44].

Although increased digest viscosity has generally been considered to be the main cause of the regulatory effects of pectin on starch degradation, other studies, type in vitro with purified pectin and pectin polysaccharides, can inhibit the enzymatic activity of amylases [45,46]. In non-gelling systems, polysaccharide–pectin and protein–amylase relationships could be driven by various non-covalent interactions, such as electrostatic, hydrophobic, and hydrogen bonds, and where the interaction may influence the spatial configuration of amylases, thus affecting their catalytic capacity [29].

The diverse studies in vivo are influenced by many experimental factors, such as subjects, diet conditions, pectin samples, feeding patterns, experimental methods, and analysis methods. Indeed, the measured changes in amylase activity may not deliver an alteration in in vivo digestion behavior, glucose uptake, or growth performance of subjects, but the changes in measured amylase activity certainly reflect a changed biochemical environment of the gastrointestinal tract or digest [29].

Several mechanisms have been proposed to explain the effect of pectin on amylase activity and glycemic response [47,48]. These include alteration of the rheology of food bolus and intestinal digest due to the swelling and gel-forming property of pectin [47,48]. The effect of pectin in increasing viscosity produces a significant delay in gastric emptying [48]. Due to limited diffusion and changed gastrointestinal transit time by dietary pectin, digestible carbohydrates in the food bolus become less available to the intestinal enzymes. This limits the rapid surge of glucose in the bloodstream, while generating a glycaemic response over a longer time. The presence of undigested carbohydrates (starch) or digested (glucose) in the small intestine is associated with gut hormone-induced responses for delayed gastric emptying [49], as well as reduced intestinal enzyme secretion [50]. High viscosity in the gut slows down the diffusion rate of digestive enzymes and substrates, thus hindering their effective interaction at the mucosal surface. The esterification of pectin is a vital factor that significantly influences the inhibition of amylase activity [51].

## 3. Pectin, Microbiota, and Production of Short-Chain Fatty Acids

### 3.1. Impact of Dietary Fiber on Gut Microbiota

The gut microbiota is the group of diverse microorganisms, including bacteria, yeast, archaea, etc., that live in the gastrointestinal tract. The composition of the microbiota varies from site to site, and the gut microbiota is considered to be the most important in regulating human health [52]. The symbiosis between the microbiota and the host contributes to homeostasis and the regulation of immunological function. On the contrary, the dysbiosis of gut microbiota leads to dysregulation of bodily functions and diseases, including cardiovascular diseases, cancers, and obesity [53].

Dietary habits are crucial for modulating the composition and function of the gut microbiome [54]. Different types of dietary fiber, mainly pectin, have received considerable interest related to their microbiota-dependent effects on host health and certain diseases [54]. The composition of the gut microbiome is important because of its beneficial effects on health [55]. The main end products of intestinal bacterial fermentation of pectin, short-chain fatty acids (SCFAs), are regulators of gut homeostasis [56]. These microbial changes induced by pectin and the product of microbial fermentation have a considerable impact on the immune system and, therefore, on the prevention and treatment of diseases [57].

Gut microbiota is the main source of bacteria, producing SCFAs through the degradation of pectin and resistant starches [58]. Interestingly, the concentration of SCFA fluctuates throughout our life, and these longitudinal changes appear to be related to the composition of our gut microbiota, which also varies during our life cycle [58,59]. Notably, the variety of our diet, which changes during our life, has a heavy influence on the quantity of SCFAs released in the intestine, modulating the amount of substrate sources for SCFAs-producing bacteria [59].

Recently, Zhang et al. [60] have reported that different structure pectins selectively promote the growth of gut microbiota that prefer rhamnogalacturonan I- and degree of esterification preferring bacteria. The structure–activity relationship of pectin is vital to understanding its physiological functions. Fermentation of polysaccharide rhamnogalacturonan I produces SCFAs, which are important in regulating the health of the host and the modulation of the diversity of intestinal microbiota bacteria. However, the activity of pectin is associated with the degree of esterification of its structure; therefore, physiological activity is modified. This knowledge provides the theoretical basis that could be used to design fiber-rich diets that target specific beneficial bacteria and their application in medical science [60].

### 3.2. Production of SCFAs

The effect of dietary fiber on the human gastrointestinal microbiota is a complex phenomenon that is essential for promoting homeostasis and influencing the regulation of human health [61]. Within this context, dietary fibers, particularly pectin, emerged as vital components, offering a captivated and varied contribution to human health. Although pectin cannot be digested by humans, it is fermented by beneficial bacteria in the large intestine via carbohydrate-active enzymes (CAZymes) [62,63]. The production of CAZymes by gut bacteria is an adaptive response to dietary pectin. CAZymes are responsible for breaking down glycosidic bonds within both carbohydrate and non-carbohydrate structures. Several classes of CAZymes include glycoside hydrolases, polysaccharide lyases, carbohydrate esterases, glycosyl transferases, carbohydrate binding modules (CBMs), and auxiliary activities [64,65]. In recent years, breakdown products of pectin, also known as pectin oligosaccharides, have gained significant attention as prebiotics because of their potential to alter microbiome composition [63]. The beneficial properties extend from gastrointestinal regulation to potential roles in allergy and inflammatory disease prevention, as well as in cancer therapy [66]. This surge in interest has led to extensive research efforts exploring the interplay between pectin and the gut microbiota.

Specific groups of gut bacteria have developed the genetic capacity to produce a diverse array of CAZymes that target different components of pectin. Fermentation of pectin results in the production of short-chain fatty acids (SCFAs), including acetate, propionate, and butyrate [58,67]. SCFAs confer various beneficial effects on the host gastrointestinal tract, including serving as an energy source for colonocytes, mitigating inflammation, and preserving intestinal barrier function [58,67]. Moreover, SCFAs have been associated with numerous health benefits throughout the body and modulate various physiological pathways, such as the immune, endocrine, vagal, and energy metabolism [68]. Additionally, SCFAs have been suggested to play a vital role in communication along the microbiota–gut–heart axis [69].

The SCFAs are saturated fatty acids with fewer than six carbon atoms. In humans, the SCFAs are produced by gut bacterial fermentation of dietary fibers or resistant starch and rapidly absorbed by epithelium to generate ATP and provide energy for colonocytes [70,71]. Although the principal locus of impact of SCFAs is the intestinal milieu, they can traverse the intestinal epithelium and the portal vein, eventually entering the systemic circulation, albeit at diminished concentration. SCFAs are the major products of anaerobic fermentation of non-digestible polysaccharides such as dietary fiber and resistant starch produced by the microbiota in the large intestine. They are composed mainly of acetate (2 carbon atoms), propionate (3 carbon atoms), and butyrate (4 carbon atoms) in an approximate molar ratio of 60:20:20, respectively [72,73]. The daily production of SCFAs, approximately 500–600 mmol, depends on dietary fiber content, the composition of gut microbiota, intestinal transit time, and the genotype of the host [74,75]. A decreased production of SCFAs is associated with metabolic diseases [76]. Furthermore, SCFAs are strongly associated with a variety of diseases, including gastrointestinal disorders, obesity, diabetes, inflammation, kidney disease, cancer, neurological disorders [77,78], and recently cardiovascular diseases [79].

### 3.3. SCFAs and Gut–Heart Axis

The gut–heart axis is related to the interaction between the gastrointestinal tract and the cardiovascular system, with SCFAs playing a relevant role in this communication. The most important mechanisms of SCFA action in the gut–heart axis involve G-protein coupled receptor activation, histone deacetylase inhibition, and mitochondrial function restoration [78]. These mechanisms contribute to cardiac function regulation and cardiovascular health maintenance. Heart failure-induced gut hypoperfusion and congestion lead to dysbiosis, reduced SCFAs production, and increased intestinal permeability [79].

## 4. Pectin and Health Benefits

### 4.1. Anti-Hyperglycemic Activity

The potential of pectin to regulate both starch digestion and postprandial hyperglycemia may be achieved by different mechanisms: inhibiting the activity of some enzymes, inducing physicochemical changes in digesta, and regulating metabolic pathways.

Pectin may limit the release of glucose in the gastrointestinal tract. It is accepted that fasting hyperglycemia is a good indicator of diabetes, and the change in postprandial blood glucose levels is a good measure of the metabolic processes of type 2 diabetes. Therefore, maintaining glucose levels within normal ranges is of great interest for health and nutrition. The ingestion of pectin, as a food additive or as an ingredient in whole foods, may have regulatory effects on postprandial blood glucose levels, fasting glucose levels, and the in vivo digestion of starch-rich foods, both in humans and animals.

This is particularly important, considering that the short, intermittent bursts of hyperglycemia may have detrimental effects on several organ systems, including the vasculature and the hematopoietic system, collectively contributing to the state of elevated CVD risk in diabetes [80].

### 4.2. Anti-Hyperlipidemic Activity

**Figure 2:**
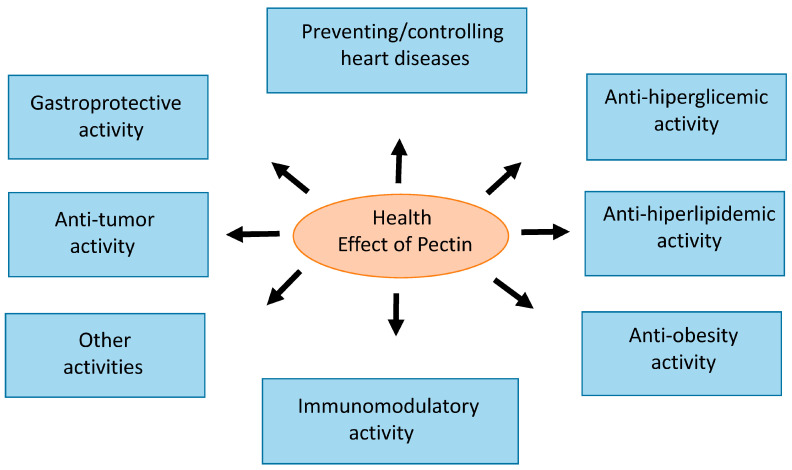
Health effects of pectin.

Interestingly, in addition to its antihyperglycemic effects, rhamnogalacturonan I (Figure 2) also showed a significant antihyperlipidemic effect in experimental animals with hyperlipidemia induced by a high-fat diet. After 30 days of treatment with rhamnogalacturonan I, fat deposition in the liver was reduced, and the ratio of monounsaturated fatty acids to saturated fatty acids changed, which suggested that pectin was able to normalize fatty acid metabolism [80,81].

### 4.3. Anti-Obesity Effect

The analysis based on body mass index results showed that pectin induced a greater lowering of glucose and insulin response in subjects with normal body weight than in overweight and obese subjects [82]. In addition, microbiota sequencing demonstrated an increase in the ratio of Bacteroidetes to Firmicutes in normal weight subjects compared to overweight subjects.

It is widely accepted that gut microbiota has a crucial role in the development of obesity. Pectin containing rhamnogalacturonan I regions can modulate the composition of obesity-related intestinal microbiota and increase the production of fatty acid butyrate, which is a dominant protective agent against obesity. When combined with the prebiotic Bifidobacterium longum BB-46, citric pectin plays the predominant role [83].

### 4.4. Preventing/Controlling Heart Disease

Cardiovascular diseases, such as hypertension, coronary artery disease, and stroke, are the most prevalent causes of morbidity and mortality worldwide. Among cardiovascular diseases, heart failure (HF) is the end result of many of them. Although there are innovations in the equipment and medical treatment of HF, the functionality and quality of life have not improved in patients

The effect of pectin on lipid metabolism and, in particular, on low-density lipoprotein is one of the most important facts to prevent and control heart disease. Observational studies have determined the effect of Gal-3 (a carbohydrate-binding protein with multiple functions) on the extracellular matrix synthesis of cardiac fibroblasts, suggesting a possible mechanism. Researchers have recently investigated Gal-3 modulation of oxidative stress to promote cardiac inflammation and fibrosis [84]. New research has demonstrated that Gal-3 regulates cell growth, proliferation, and apoptosis through cell–cell and cell–matrix interactions. Its importance is highlighted in the development and progression of cardiovascular diseases, and its significance increases in patients with heart failure (HF). In atherosclerosis, Gal-3 promotes the recruitment of monocytes to the arterial wall, increasing inflammation and atheroma. In acute myocardial infarction (AMI), Gal-3 expression increases in infarcted and remote areas from the onset of AMI, playing a key role in macrophage infiltration, differentiation to the M1 phenotype, inflammation, and interstitial fibrosis through collagen synthesis. Gal-3 is a potential therapeutic target for the treatment of cardiovascular diseases and the management of cardiac deterioration [85]. Raw hawthorn pectin from hawthorn (RHP) is high methoxyl pectin, which is able to protect against injury induced by AMI. After the stir-frying of hawthorn, pectin from stir-fried hawthorn (FHP) transformed into low methoxyl pectin. The results of this study revealed that RHP and FHP played a protective role in myocardial ischaemia by regulating intestinal flora and SCFAs [86].

### 4.5. Butirate in Regulating the Myocardial Ischemia–Reperfusion Injury (MIRI)

Acute myocardial infarction is an important cause of death worldwide. Reperfusion is a required event that induces a further process called myocardial ischemia/reperfusion (I/R) injury, which includes inflammation, oxidative stress, and apoptosis, and induces cardiomyocyte damage [87]. However, the prevention and treatment of myocardial I/R injury remain limited.

Sodium butyrate is absorbed by the intestinal mucosal cells and enters the bloodstream, then it goes to the liver and participates in the tricarboxylic acid (TCA) cycle. In this cycle, butyrate reacts with other metabolites to produce energy. Butyrate not only provides energy but also affects energy homeostasis and participates in the process of cell growth and differentiation. It is absorbed by the intestinal mucosal cells and enters the bloodstream, then goes to the liver and participates in the TCA cycle, where it reacts with other metabolites to produce energy [88]. Moreover, previous studies by Hu et al. showed that pre-treatment with sodium butyrate significantly reduced myocardial infarct size, consistent with decreased lactate dehydrogenase and creatine kinase levels, biomarkers of tissue damage. In addition, sodium butyrate was found to significantly inhibit MIRI-induced expression of TNF-α, IL-6, and HMGB1 [89].

Moreover, accumulating studies suggested that the gut–brain neural mechanism may also contribute to the beneficial effects of butyrate [90]. As a key member of the gut–brain axis, the vagus nerve innervates many gastrointestinal organs in addition to cardiopulmonary organs [91]. In this respect, Yu et al. conducted a series of studies on the relationship between butyrate and the vagus nerve, in which observed that butyrate treatment significantly ameliorated myocardial I/R injury via a gut–brain neural circuit, and that this cardioprotective effect was probably mediated by suppression of the sympathetic nervous system [92].

### 4.6. Anti-Tumor Activity

Many studies have demonstrated that rhamnogalacturonan I, as well as its synthesized branches, can effectively suppress the growth of cancer cells through tumor growth inhibition, anti-metastasis activity, regulating gene expression, and immunological systems [93,94,95]. Nowadays, several approaches have been developed to enrich rhamnogalacturonan I regions in pectin and enhance the anti-tumor activities of these pectin fractions. Modified citrus pectin, a Gal binder, was employed as a new radiosensitizing agent for ionizing radiation, suggesting yet another application for RG-1 pectin [96]. However, more systematic investigations of in vivo studies are needed to better understand the potential mechanism of antitumor activity by RG-I pectin [97].

### 4.7. Gastroprotective Activity

Specific structures of modified pectin can protect the digestive system and ameliorate gastric diseases. Dietary fibers cover a broad array of rhamnogalacturonan I forms and have been proposed to improve the status of the colonic mucus barrier, maintain intestinal integrity, resist bacterial invasion, and prevent inflammation. On the other hand, dietary fiber deprivation results in damage to the mucus barrier by microbiota and increases pathogen susceptibility [29].

### 4.8. Immunomodulatory Activity

A great number of rhamnogalacturonan I domains have been reported with potent immunological properties, particularly towards macrophages, lymphocytes, and the complement system [86].

## 5. Discussion

Although the mode of action of dietary fiber and pectin in the consumer’s body is not fully understood, much knowledge has been gained in the last five years, especially about the mechanism of pectin and its effects on health [86,98].

The average daily intake of pectin from fruits and vegetables has been estimated to be around 5 g considering a fruit and vegetable consumption of 500 g per day [31]. However, the real fruit and vegetable consumption in most countries is less than 200 g per day [99]. The pectin contents in many fruits and vegetables range from 0.1% to 2.5% on a wet basis. To improve health, the European Food Safety Authority recommends a daily intake of 30 g of pectin, with benefits such as reducing postprandial glycemia, maintaining cholesterol at normal levels, and increasing satiety, which leads to a decrease in calorie intake [100].

For all these reasons and based on scientific data, it is necessary to increase F&V consumption to prevent chronic diseases and improve population health and quality of life.

Some simple recommendations are proposed throughout the world by the “5 a day campaign”. This means that a person must eat at least one fruit at breakfast; one salad and one fruit at lunch, and another salad and fruit at dinner. If the average weight of a fruit or a salad is 80 g, with the five portions of F&V per day, a person can obtain the minimum of 400 g per day recommended by FAO and WHO [9]. In recognition of the critical role F&Vs play in promoting both healthy diets and sustainable food systems, the United Nations declared 2021 the International Year of Fruits and Vegetables (IYFV 2021) [101,102].

## 6. Recommendations

To achieve this objective, it is very important to educate children from a young age at home and at school about the need to consume fruit and vegetables with activities such as accompanying parents to buy at free fairs and supermarkets, as an entertaining family activity. To participate in the preparation of fruit salads and desserts in the home kitchen, uncooked, if possible, because they lose their fiber and antioxidant benefits. When snacks are sent to school, preferably fruit, and during festivities, we always put salads and fruits together with the other foods at the celebration. And one important recommendation for pectin is to encourage children to eat fruits, where possible, with peel, where the greatest amount of fiber is pectin. Health promotion programs like social marketing programs can help spread concepts of healthy eating, including the promotion of fruit and vegetable consumption. Simpler population-level intervention options, such as providing vouchers for free fruits and vegetables to low-income families, or distributing more fruits and vegetables in schools, have proven to be more effective than high government commitment approaches, such as nutritional labeling, taxes, or mass media campaigns, which most governments tend to favor because they are easier to implement [103].

## Data Availability

Not applicable.
